# Impact of Racial and Socioeconomic Disparities on Access to Interspinous Spacer for Treatment of Lumbar Spinal Stenosis: A Nationwide Medicare Analysis

**DOI:** 10.1007/s40615-024-02097-8

**Published:** 2024-07-23

**Authors:** Annie M. Cho, Oth Tran, Alysha M. McGovern, Kheng Sze Chan, Robert Jason Yong

**Affiliations:** 1 Brigham and Women’s Center for Pain Medicine, Chestnut Hill, MA USA; 2 Health Economics and Outcomes Research, Boston Scientific Corporation, Marlborough, MA USA; 3 University of California, Los Angeles Medical Center, Los Angeles, CA USA

**Keywords:** Lumbar spinal stenosis, Interspinous spacers, Health equity, Social determinants of health, Racial bias

## Abstract

**Background:**

In mild to moderate lumbar spinal stenosis (LSS) where conservative care treatments fail, minimally invasive treatments, such as interspinous spacers without decompression or fusion (ISD), may be appropriate. While previous studies have demonstrated racial and socioeconomic disparities in the surgical treatment of LSS, there are limited data on how those factors impact accessibility to these procedures. This study explored demographic, socioeconomic, and geographic differences in the use of ISD.

**Methods:**

Using the Medicare 100% files from 2017 through 2022, this retrospective claims analysis identified when and if patients diagnosed with LSS received ISD implantation. Cox proportional hazards regression was used to examine the association between racial and socioeconomic characteristics and the rate of ISD implantation, stratified by geographic region.

**Results:**

A total of 1,316,622 individuals met the inclusion criteria; 4730 (0.4%) underwent ISD implantation, with a mean (standard deviation) time to treatment of 11.9 (13.2) months after diagnosis. The likelihood of ISD implantation was higher for older patients (except for the oldest group), males, those with lower disease burden, and White patients. Cox regression revealed that the associations of racial and socioeconomic factors with ISD implantation varied by U.S. region. In the Midwest and Northeast, lower median household income was associated with a decreased likelihood of ISD implantation regardless of race, while in the South, Black patients were less likely to undergo ISD implantation regardless of income.

**Conclusions:**

The observed disparities in access to ISD implantation mirror existing trends in surgical interventions for LSS, suggesting further study and interventions are needed to address inequities.

**Supplementary Information:**

The online version contains supplementary material available at 10.1007/s40615-024-02097-8.

## Introduction

Individuals with lumbar spinal stenosis (LSS) often experience pain or weakness in the legs and/or difficulty walking [1] and suffer significant decrements in their health-related quality of life [2]. A variety of treatments are available for individuals with LSS. In the case of failed conservative care treatments, such as pain medications, physical therapy, and epidural injections [3, 4], some patients may benefit from surgical interventions, which may include the placement of interspinous spacers, direct or indirect decompression of the spinal canal with or without fusion, or traditional spinal surgery in order to decompress the spinal canal and retain stability [3].

For those with mild to moderate LSS for whom open spinal surgery may pose a significant risk, minimally invasive treatments such as interspinous spacers may be appropriate [5, 6]. The interspinous spacer without decompression or fusion (ISD) is a minimally invasive procedure that is designed to provide long-term relief of back and leg pain for LSS patients [7].

Previous studies have demonstrated racial and socioeconomic disparities in the surgical treatment of LSS. The rate of surgical hospitalization among non-Hispanic White patients with LSS has been shown to be significantly higher than those of either non-Hispanic Black or Hispanic ethnicity [8]. Additionally, Black patients are less likely to undergo spinal surgery to treat lower back pain [9]. Postoperatively, Black patients have longer lengths of stay and more often experience complications and re-admission after surgical treatment of LSS in relation to their White counterparts [10–12]. Length of stay and post-operative outcomes have also been shown to differ by socioeconomic status and insurance type [13–15].

These previous studies have highlighted disparities in outcomes after spinal surgery. However, the impact of racial and socioeconomic factors on accessibility to minimally invasive procedures for the treatment of LSS has not yet been explored. Therefore, this study aimed to characterize the demographic, socioeconomic, and geographic differences in the use of ISD among the Medicare-insured population with LSS. Identifying and understanding disparities in access to these types of procedures within this population is a crucial step toward ensuring equitable care and improving outcomes for those with LSS. The results have the potential to inform interventions and policies to address disparities and enhance access to minimally invasive treatment options when appropriate.

## Methods

### Study Design, Data Source, and Study Population

This was a retrospective claims analysis using the Medicare 100% Standard Analytical Files (SAFs) from 2017 through 2022. These files include enrollment, demographic, and encounter data for Medicare beneficiaries, and reflect encounters that occur in either the inpatient or outpatient setting. Patients were identified using the Tenth Revision of the International Classification of Disease (ICD-10) diagnosis codes for LSS (M48.061 or M48.062) on Medicare claims. Eligible patients had at least two claims (from either the inpatient or outpatient setting) with a LSS diagnosis between 1/1/2017 and 12/31/2022. The index date was defined as the date of the first claim with a LSS diagnosis.

Patients aged less than 50 years on the index date were excluded to omit patients with congenital LSS. Also excluded were those with less than 12 months of data available prior to the index date (designated as the baseline period needed to identify comorbidity burden and prior encounters) and those with any ISD procedures or other lumbar spine surgeries, such as open decompression, laminectomy, laminotomy, other interspinous spacer implantation, fusion, minimally invasive lumbar decompression (MILD®), or other interventions (codes are available in the Supplemental Material).

### Measures and Outcomes

Demographic information and geographic location were obtained from enrollment data. County-level median household income from the United States (U.S.) Census Bureau Small Area Income and Poverty Estimates (SAIPE) [16] dataset was cross-referenced with patients’ geographic location (county code) from the SAFs for use as a proxy for patient income; for analysis, household income was classified into quartiles. Comorbidity burden was established using medical diagnoses during the baseline period, and the Charlson Comorbidity Index (CCI) [17], an indicator of comorbidity severity, was calculated using medical claims during the baseline period. The primary outcomes were implantation of ISD during follow-up and the time in months to implantation of ISD.

### Statistical Analysis

Demographic, geographic, and clinical characteristics were summarized using means and standard deviations (SDs) for continuous variables or counts and percentages for categorical variables. To examine the association between racial and socioeconomic characteristics and the rate of ISD implantation, Cox proportional hazards regression was used. To explore the interaction effects between geographic region and household income, Cox regressions were stratified by region.

## Results

**Fig. 1:**
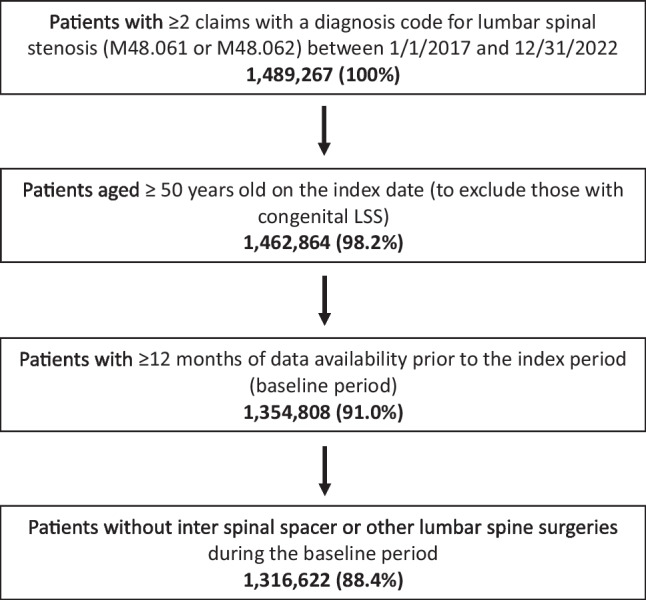
Patient selection

From a total of 1,489,267 patients with two or more LSS claims during 2017–2022, a total of 1,316,622 (88.4%) patients met the inclusion/exclusion criteria (Fig. 1). The mean (SD) age of eligible patients was 74.0 (8.5) years; 57% were female and 88.5% were White (Table 1). Most patients lived in the South (35.5%) or Midwest (29.3%) regions of the U.S., and the most common comorbid conditions were hypertension (59.3%), osteoarthritis (32.1%), and diabetes (25.9%) (Table 1). Over half of Black patients resided in the South (54.2%), while a third of White patients lived in the South (34.6%) or Midwest (30.3%). On average, Black patients had higher CCI (1.7) compared to White patients (1.2) (Table 1). A total of 4730 patients (0.4%) underwent ISD implantation and the mean (SD) time to treatment was 11.9 (13.2) months (Table 1). The mean (SD) CCI ranged from 1.2 (1.8) among patients in the highest income quartile to 1.3 (1.8) among patients in the lowest income quartile.

**Table 1:** Patient characteristics

	**All patients**		**White patients**		**Black patients**	
	***N*/mean**	**%/SD**	***N*/mean**	**%/SD**	***N*/mean**	**%/SD**
**Total**	1,316,622	100%	1,165,586	100%	83,752	100%
**Demographics at index diagnosis**						
Age at index (mean, SD)	74.0	8.5	74.0	8.4	70.6	9.2
Sex (*N*, %)						
Female	748,856	56.9%	659,454	56.6%	54,547	65.1%
Male	567,766	43.1%	506,132	43.4%	29,205	34.9%
Region (*N*, %)						
Midwest	385,839	29.3%	352,735	30.3%	20,271	24.2%
Northeast	223,637	17.0%	202,444	17.4%	10,674	12.7%
South	467,196	35.5%	403,852	34.6%	45,370	54.2%
West	238,114	18.1%	205,134	17.6%	7259	8.7%
Other/unknown	1836	0.1%	1421	0.1%	178	0.2%
Race (*N*, %)						
White	1,165,586	88.5%				
Black	83,752	6.4%				
Other/unknown	67,284	5.1%				
Follow-up length in months (mean, SD)	37.2	16.9	37.1	16.9	38.4	17.0
Median household income based on zip code (*N*, %)	1,315,118		1,164,449		83,587	
Quartile 1 (< $54,815)	328,160	25.0%	291,388	25.0%	25,102	30.0%
Quartile 2 ($54,815–$64,235)	327,880	24.9%	294,330	25.3%	19,939	23.9%
Quartile 3 ($64,236–$75,624)	335,415	25.5%	295,410	25.4%	21,214	25.4%
Quartile 4 (> $75,624)	323,663	24.6%	283,321	24.3%	17,332	20.7%
**Charlson Comorbidity Index during baseline (mean, SD)**	1.3	1.8	1.2	1.8	1.7	2.1
**Clinical comorbid conditions during baseline**						
Atrial fibrillation	181,683	13.8%	168,128	14.4%	7,505	9.0%
Asthma	97,935	7.4%	82,354	7.1%	10,378	12.4%
Back syndrome	26,949	2.0%	24,035	2.1%	1920	2.3%
Chronic obstructive pulmonary disease	163,081	12.4%	146,133	12.5%	11,391	13.6%
Diabetes	340,629	25.9%	285,827	24.5%	33,482	40.0%
Diabetic neuropathy	99,078	7.5%	82,600	7.1%	10,586	12.6%
Hypertension	780,533	59.3%	685,642	58.8%	57,274	68.4%
Congestive heart failure	147,835	11.2%	128,983	11.1%	13,001	15.5%
Obesity	195,376	14.8%	167,417	14.4%	19,241	23.0%
BMI > 40	50,391	3.8%	42,341	3.6%	6091	7.3%
Osteoporosis	134,036	10.2%	121,779	10.4%	5271	6.3%
Osteoarthritis	422,471	32.1%	371,458	31.9%	31,404	37.5%
Lumbar spondylolisthesis	64,473	4.9%	57,284	4.9%	3970	4.7%
Spondylolisthesis in other spinal regions	30,618	2.3%	27,478	2.4%	1695	2.0%
Intervertebral disc disorders	193,366	14.7%	172,743	14.8%	11,972	14.3%
Vascular claudication	21,031	1.6%	18,520	1.6%	1650	2.0%
**Outcomes**						
ISD during the follow-up period	4730	0.4%	4348	0.4%	209	0.2%
Months from the index diagnosis to ISD procedure	11.9	13.2	11.8	13.2	13.8	14.5

*BMI* body mass index, *ISD* Vertiflex interspinous spacer without decompression or fusion, *SD* standard deviation

An adjusted Cox model found that region, race, income level, and CCI played a significant role in access to an ISD. Specifically, the likelihood of ISD implantation was higher for older patients (except for the oldest age group), slightly higher for male than female patients, lower for patients with higher CCI, and significantly lower for Black patients than White patients (Table 2). Compared with the highest household income quartile, patients in the lowest quartile had a significantly higher likelihood of undergoing ISD. Additionally, patients in the Northeast and West were significantly less likely to undergo ISD than those in the Midwest.

**Table 2:** Cox proportional hazard model: time to ISD procedure

Independent variables	Hazard Ratio	95% confidence interval		
		**Lower**	**Upper**	***p* value**
Age group (reference, 50–64)				
65–69	1.139	1.011	1.282	**0.032**
70–74	1.237	1.101	1.390	** < 0.001**
75–79	1.366	1.213	1.538	** < 0.001**
80–84	1.415	1.247	1.605	** < 0.001**
> = 85	0.908	0.784	1.053	0.202
Sex (reference, female)	1.098	1.035	1.165	**0.002**
**Race (reference, White)**				
Black	0.658	0.571	0.757	** < 0.001**
Other/unknown	0.772	0.662	0.900	**0.001**
**Region (reference, Midwest)**				
Northeast	0.743	0.674	0.819	** < 0.001**
South	1.014	0.948	1.085	0.681
West	0.706	0.642	0.777	** < 0.001**
Missing	0.000			
**Index year (reference, 2017)**				
2018	1.064	0.953	1.187	0.270
2019	1.607	1.438	1.796	** < 0.001**
2020	2.272	2.026	2.547	** < 0.001**
2021	2.274	2.016	2.565	** < 0.001**
2022	2.528	2.084	3.066	** < 0.001**
**Household income (reference, quartile 4)**				
Quartile 1	1.596	1.465	1.738	** < 0.001**
Quartile 2	1.003	0.915	1.100	0.944
Quartile 3	1.014	0.927	1.110	0.757
**Charlson Comorbidity Index**	0.933	0.909	0.956	** < 0.001**
**Comorbid conditions**				
Asthma	1.073	0.955	1.205	0.236
Back syndrome	1.207	1.007	1.446	**0.042**
Chronic obstructive pulmonary disease	1.116	1.015	1.227	**0.024**
Diabetes	1.118	1.039	1.202	**0.003**
Diabetic neuropathy	0.782	0.684	0.894	** < 0.001**
Intervertebral disc disorders	1.375	1.276	1.483	** < 0.001**
Congestive heart failure	1.112	0.987	1.253	0.081
Hypertension	1.099	1.031	1.171	**0.004**
Obesity	0.969	0.883	1.064	0.514
Osteoarthritis	1.049	0.984	1.118	0.144
Osteoporosis	0.833	0.748	0.927	**0.001**
Spondylolisthesis in other spinal regions	0.671	0.535	0.841	**0.001**
Lumbar spondylolisthesis	0.920	0.802	1.055	0.231
Vascular claudication	1.167	0.943	1.445	0.156
BMI > 40	0.903	0.758	1.076	0.253

*BMI* body mass index

Bold entries are statistically significant at *p *<0.05

To delineate further how race and income level interact with region on access to ISD, separate Cox models were conducted among a subgroup of patients living in the South, Midwest, and Northeast (Table 3). A Cox model was not conducted for the subgroup of patients in the West due to the low sample size of Black patients (*N* = 8) overall and zero Black patients in the lower income quartiles. The adjusted Cox model found that in the South, patients in the lower income quartiles (Q1, Q2, and Q3) were more likely to undergo ISD implantation, but Black patients were less likely (Table 3). In the Midwest, race did not play a significant role. However, income level significantly predicted ISD implantation. Patients in lower income quartiles were less likely to undergo ISD implantation compared with patients in the highest quartile. Lastly, patients in the Northeast demonstrated a similar pattern to the results of patients residing in the Midwest: patients in the lower income quartiles were less likely to receive an ISD compared to those in the highest quartile.

**Table 3:** Cox proportional hazard models, stratified by geographic region: time to ISD procedure

Independent variables	Region, South				Region, Midwest				Region, Northeast			
	**Hazard Ratio**	**Lower CL**	**Upper CL**	***p* value**	**Hazard ratio**	**Lower CL**	**Upper CL**	***p* value**	**Hazard Ratio**	**Lower CL**	**Upper CL**	***p* value**
**Age group (reference, 50–64)**												
65–69	1.143	0.959	1.363	0.136	1.032	0.844	1.262	0.757	1.418	0.970	2.072	0.071
70–74	1.148	0.964	1.366	0.121	1.243	1.021	1.511	**0.030**	1.425	0.982	2.067	0.062
75–79	1.278	1.070	1.527	**0.007**	1.228	1.004	1.503	**0.046**	1.809	1.249	2.622	**0.002**
80–84	1.389	1.149	1.680	**0.001**	1.103	0.885	1.376	0.382	2.114	1.444	3.094	** < 0.001**
> = 85	0.908	0.722	1.140	0.405	0.784	0.608	1.012	0.061	1.124	0.730	1.732	0.595
**Sex (reference, female)**	1.130	1.031	1.238	**0.009**	1.007	0.907	1.119	0.892	1.287	1.089	1.520	**0.003**
**Race (reference, White)**					1.000				1.000			
Black	0.469	0.381	0.578	** < 0.001**	1.160	0.931	1.446	0.186	0.671	0.418	1.076	0.098
Other/unknown	0.610	0.453	0.820	**0.001**	0.734	0.536	1.007	0.055	0.624	0.394	0.989	**0.045**
**Household income (reference, Q4)**					1.000				1.000			
Quartile 1	4.444	3.623	5.452	** < 0.001**	0.762	0.658	0.883	** < 0.001**	0.518	0.366	0.735	** < 0.001**
Quartile 2	2.519	2.030	3.124	** < 0.001**	0.512	0.440	0.597	** < 0.001**	0.695	0.531	0.909	**0.008**
Quartile 3	1.808	1.436	2.275	** < 0.001**	0.745	0.644	0.860	** < 0.001**	1.111	0.928	1.331	0.252
**Index year (reference, 2017)**	1.000				1.000				1.000			
2018	1.463	1.218	1.756	** < 0.001**	0.893	0.742	1.075	0.232	0.736	0.553	0.981	**0.037**
2019	2.088	1.737	2.510	** < 0.001**	1.483	1.230	1.789	** < 0.001**	1.191	0.890	1.593	0.240
2020	2.386	1.970	2.892	** < 0.001**	2.444	2.017	2.961	** < 0.001**	1.794	1.326	2.426	** < 0.001**
2021	2.140	1.746	2.624	** < 0.001**	2.701	2.210	3.301	** < 0.001**	2.127	1.559	2.901	** < 0.001**
2022	1.936	1.378	2.722	** < 0.001**	3.846	2.825	5.236	** < 0.001**	3.390	2.135	5.384	** < 0.001**
**Charlson Comorbidity Index**	0.922	0.886	0.960	** < 0.001**	0.933	0.893	0.975	**0.002**	0.931	0.869	0.997	**0.042**
**Comorbid conditions**					1.040	0.847	1.276	0.711	0.976	0.694	1.371	0.887
Asthma	1.184	0.994	1.410	0.059	1.184	0.874	1.603	0.275	0.793	0.393	1.598	0.516
Back syndrome	1.245	0.958	1.618	0.101	0.983	0.829	1.166	0.845	0.948	0.701	1.283	0.729
Chronic obstructive pulmonary disease	1.329	1.160	1.523	** < 0.001**	1.174	1.036	1.330	**0.012**	1.060	0.857	1.312	0.590
Diabetes	1.107	0.990	1.236	0.074	0.865	0.694	1.077	0.195	0.948	0.643	1.396	0.785
Diabetic neuropathy	0.721	0.586	0.889	**0.002**	1.469	1.294	1.668	** < 0.001**	1.481	1.179	1.859	**0.001**
Intervertebral disc disorders	1.390	1.239	1.558	** < 0.001**	1.038	0.842	1.280	0.724	1.109	0.788	1.562	0.553
Congestive heart failure	1.122	0.934	1.349	0.218	1.026	0.918	1.147	0.649	0.859	0.722	1.023	0.087
Hypertension	1.262	1.141	1.395	** < 0.001**	0.933	0.798	1.092	0.390	1.176	0.899	1.537	0.236
Obesity	0.952	0.822	1.102	0.509	1.096	0.982	1.224	0.101	0.945	0.783	1.140	0.554
Osteoarthritis	1.051	0.952	1.161	0.322	0.787	0.654	0.947	**0.011**	0.837	0.617	1.136	0.253
Osteoporosis	0.845	0.714	1.000	0.050	0.681	0.469	0.987	**0.042**	0.818	0.448	1.497	0.515
Spondylolisthesis in other spinal regions	0.563	0.379	0.838	**0.005**	1.051	0.845	1.308	0.653	0.921	0.620	1.369	0.685
Lumbar spondylolisthesis	0.892	0.711	1.118	0.320	1.373	0.979	1.926	0.066	0.780	0.369	1.648	0.515
Vascular claudication	1.100	0.789	1.533	0.574	0.807	0.605	1.077	0.146	1.305	0.821	2.072	0.260
BMI > 40	0.944	0.718	1.243	0.683	1.032	0.844	1.262	0.757	1.418	0.970	2.072	0.071

*BMI* body mass index, *CL* 95% confidence limit

Bold entries are statistically significant at *p* <0.05

## Discussion

In this analysis, we observed significant associations between ISD implantation and several patient characteristics, including age, sex, race, and median household income. The fact that older patients were more likely to receive an ISD may be a reflection of surgical risk, namely that older patients more often had contraindications to surgical interventions that made minimally invasive procedures a better choice. Comorbidities associated with ISD placement included chronic obstructive pulmonary disease, diabetes, and hypertension, which could signal generally poor health and an increased surgical risk. Additionally, some of these associations varied by U.S. census region. In the Midwest and Northeast, after adjusting for race, patients in the lowest median income quartile were less likely to undergo ISD implantation. A partial explanation could be that patients in the lower income quartile had a higher comorbidity burden, which was associated with less likelihood of ISD implantation, as observed in the multivariate results. Uniquely, in the South, after adjusting for race, patients in the lower income quartiles were more likely to undergo ISD implantation compared to those in the highest quartile. However, Black patients in the South were significantly less likely to undergo ISD implantation regardless of income level. This may partly be because Black patients had a higher comorbidity burden compared to White patients. It may also be a function of systematic bias. Others have explored the existence of racial bias in the assessment and management of pain [18, 19], as well as in the receipt of certain pain treatments [20]. The presence of systemic bias in the healthcare system or implicit provider bias stemming from unconscious attitudes or stereotypes can contribute to discriminatory practices within a healthcare institution. Lack of trust and perceptions of discrimination may even cause some individuals of color to avoid care [18]. However, from the current data, we cannot determine if a patient was offered an ISD implantation and declined, so we are unable to examine if this occurred within the study cohort.

Much of the existing literature examines disparities in outcomes following LSS surgery. These have shown significant differences in lengths of stay, complications, and readmissions by race [10–12, 21] and socioeconomic status or insurance type [13–15]. However, Skolasky et al. (2013) examined trends in surgical hospitalizations for LSS from 2000 through 2009. In addition to a significant increase in the overall rate during that time period, the authors also noted that the rate for non-Hispanic White patients (1.074 per 1000 patients) was almost twice that of non-Hispanic Black patients (0.558 per 1000) and more than three times that of Hispanic patients (0.339 per 1000) [8]. The authors posit multiple possible reasons for this difference, including the possibility of differences in access to care. This study demonstrated similar findings wherein Black patients overall and lower-income patients in the Midwest and Northeast were observed to experience lower rates of ISD implantation for LSS treatment than their White and higher-income counterparts, respectively.

The strengths of this study include the large, geographically diverse sample of patients and the lengthy follow-up period to identify ISD procedures. However, the study is also subject to the limitations of claims data, namely that the SAFs are administrative in nature, and the codes for procedures and diagnoses may not reflect all relevant clinical details. Additionally, administrative data can be subject to data entry errors. Furthermore, the study population reflects only those with Medicare Fee-for-Service coverage; therefore, the results may not be generalizable to patients with other insurance plans, including Medicare Advantage, or to patients without health insurance coverage. Excluding younger patients may also limit the generalizability of the study findings to the broader population of individuals experiencing symptomatic LSS, particularly those between the ages of 40 and 64. However, despite this limitation, this analysis of Medicare-insured persons may capture and be representative of a large proportion of patients seeking treatment for LSS, given the prevalence of LSS is mostly concentrated among adults aged 65 and older.

The findings of this study highlight significant racial, socioeconomic, and geographic disparities in access to minimally invasive ISD for the treatment of LSS among the Medicare-insured population. These disparities suggest systemic issues within the healthcare system that impact equitable access to innovative treatments. Theoretically, the study underscores the importance of examining healthcare disparities through the lens of social determinants of health, including race and socioeconomic status, aligning with existing theories on health inequities. Contextually, the results advocate for policy interventions aimed at reducing barriers to accessing minimally invasive treatments, addressing potential implicit biases in clinical practice, and emphasizing the need for public health initiatives to increase awareness and access among underrepresented and underserved populations. Future research should focus on exploring the underlying causes of these disparities and evaluating the impact of targeted interventions. Understanding and addressing these disparities is crucial for achieving health equity and ensuring that all patients, regardless of race or socioeconomic status, have access to appropriate and effective treatments for LSS.

## Conclusion

Our findings indicate that in the Midwest and Northeast, higher median household income was associated with increased access to interspinous spacer treatment. However, in the South, patients in lower-income quartiles were more likely to undergo interspinous spacer implantation, highlighting regional variations in the impact of socioeconomic factors on access to ISD for LSS. Additionally, racial disparities were evident, with Black patients in the South being less likely to undergo interspinous spacer implantation compared to their White counterparts.

The observed disparities in access to interspinous spacer treatment mirror existing trends in surgical interventions for LSS, emphasizing the need for further investigation and interventions to address healthcare inequities. Our study contributes valuable insights into the nuanced interactions between race, income, and geographic region in shaping access to specific LSS treatments. Future research and policy initiatives should aim to reduce these disparities and ensure equitable access to effective interventions for individuals suffering from lumbar spinal stenosis.

## Supplementary Information

Below is the link to the electronic supplementary material.

Supplementary file 1 (DOCX 18 KB)

## References

[1] Deer T, et al. A review of lumbar spinal stenosis with intermittent neurogenic claudication: disease and diagnosis. Pain Med. 2019;20(Suppl 2):S32-s44. 10.1093/pm/pnz161. 31808530 10.1093/pm/pnz161 PMC7101166

[2] Battié MC, et al. Health-related quality of life and comorbidities associated with lumbar spinal stenosis. Spine J. 2012;12(3):189–95. 10.1016/j.spinee.2011.11.009. 22193054 10.1016/j.spinee.2011.11.009

[3] Cairns K, et al. Cost-effectiveness and safety of interspinous process decompression (Superion). Pain Med. 2019;20(Suppl 2):S2-s8. 10.1093/pm/pnz245. 31808529 10.1093/pm/pnz245 PMC6896024

[4] Diwan S, et al. An algorithmic approach to treating lumbar spinal stenosis: an evidenced-based approach. Pain Med. 2019;20(Suppl 2):S23-s31. 10.1093/pm/pnz133. 31808532 10.1093/pm/pnz133 PMC7101167

[5] Bini W, Miller LE, Block JE. Minimally invasive treatment of moderate lumbar spinal stenosis with the superion interspinous spacer. Open Orthop J. 2011;5:361–7. 10.2174/1874325001105010361. 22043255 10.2174/1874325001105010361 PMC3201565

[6] Hartman J, Granville M, Jacobson RE. The use of Vertiflex® interspinous spacer device in patients with lumbar spinal stenosis and concurrent medical comorbidities. Cureus. 2019;11(8): e5374. 10.7759/cureus.5374. 31616607 10.7759/cureus.5374 PMC6786837

[7] Scientific, B. The vertiflex procedure 2023. https://www.bostonscientific.com/en-US/medical-specialties/pain-management/the-vertiflex-procedure.html. Accessed May 2024.

[8] Skolasky RL, Maggard AM, Thorpe RJ, Wegener ST, Riley LH. United States hospital admissions for lumbar spinal stenosis: racial and ethnic differences, 2000 through 2009. Spine (Phila Pa 1976). 2013;38(26):2272–8. 10.1097/BRS.0b013e3182a3d392. 23873234 10.1097/BRS.0b013e3182a3d392

[9] Chen Q, et al. Racial and ethnic differences in the use of lumbar imaging, opioid analgesics and spinal surgery for low back pain: a systematic review and meta-analysis. Eur J Pain. 2023;27(4):476–91. 10.1002/ejp.2075. 36585947 10.1002/ejp.2075

[10] Aladdin DEH, Tangel V, Lui B, Pryor KO, Witkin LR, White RS. Black race as a social determinant of health and outcomes after lumbar spinal fusion surgery: a multistate analysis, 2007 to 2014. Spine (Phila Pa 1976). 2020;45(10):701–11. 10.1097/brs.0000000000003367. 31939767 10.1097/BRS.0000000000003367

[11] Lad SP, Bagley JH, Kenney KT, Ugiliweneza B, Kong M, Bagley CA, et al. Racial disparities in outcomes of spinal surgery for lumbar stenosis. Spine (Phila Pa 1976). 2013;38(11):927–35. 10.1097/BRS.0b013e31828165f9. 23232216 10.1097/BRS.0b013e31828165f9

[12] Sanford Z, et al. Racial disparities in surgical outcomes after spine surgery: an ACS-NSQIP analysis. Global Spine J. 2019;9(6):583–90. 10.1177/2192568218811633. 31448190 10.1177/2192568218811633 PMC6693061

[13] Hagan MJ, et al. Neighborhood-level socioeconomic status, extended length of stay, and discharge disposition following elective lumbar spine surgery. N Am Spine Soc J. 2022;12: 100187. 10.1016/j.xnsj.2022.100187. 36561892 10.1016/j.xnsj.2022.100187 PMC9763740

[14] Holbert SE, Andersen K, Stone D, Pipkin K, Turcotte J, Patton C. Social determinants of health influence early outcomes following lumbar spine surgery. Ochsner J. 2022;22(4):299–306. 10.31486/toj.22.0066. 36561097 10.31486/toj.22.0066 PMC9753941

[15] Lad SP, Huang KT, Bagley JH, Hazzard MA, Babu R, Owens TR, et al. Disparities in the outcomes of lumbar spinal stenosis surgery based on insurance status. Spine (Phila Pa 1976). 2013;38(13):1119–27. 10.1097/BRS.0b013e318287f04e. 23354106 10.1097/BRS.0b013e318287f04e

[16] United States Census Bureau. Small area income and poverty estimates (SAIPE) program. 2023. https://www.census.gov/programs-surveys/saipe.html. Accessed 15 Nov 2023

[17] Charlson ME, et al. A new method of classifying prognostic comorbidity in longitudinal studies: development and validation. J Chronic Dis. 1987;40(5):373–83. 10.1016/0021-9681(87)90171-8. 3558716 10.1016/0021-9681(87)90171-8

[18] Ghoshal M, et al. Chronic noncancer pain management and systemic racism: time to move toward equal care standards. J Pain Res. 2020;13:2825–36. 10.2147/jpr.S287314. 33192090 10.2147/JPR.S287314 PMC7654542

[19] Hoffman KM, et al. Racial bias in pain assessment and treatment recommendations, and false beliefs about biological differences between blacks and whites. Proc Natl Acad Sci U S A. 2016;113(16):4296–301. 10.1073/pnas.1516047113. 27044069 10.1073/pnas.1516047113 PMC4843483

[20] Roseen EJ, et al. Racial and ethnic disparities in the incidence of high-impact chronic pain among primary care patients with acute low back pain: a cohort study. Pain Med. 2023;24(6):633–43. 10.1093/pm/pnac193. 36534910 10.1093/pm/pnac193 PMC10233486

[21] Drazin D, et al. Racial disparities in elderly patients receiving lumbar spinal stenosis surgery. Global Spine J. 2017;7(2):162–9. 10.1177/2192568217694012. 28507886 10.1177/2192568217694012 PMC5415158

