# Supplementary material for: Impact of Racial and Socioeconomic Disparities on Access to Interspinous Spacer for Treatment of Lumbar Spinal Stenosis: A Nationwide Medicare Analysis
Source: J Racial Ethn Health Disparities. 2024 Jul 23;12(5):2896–903. doi: 10.1007/s40615-024-02097-8 (PMC12446150; doi:10.1007/s40615-024-02097-8)
Supplement: Supplementary file 1 — (DOCX 18 KB) [file 40615_2024_2097_MOESM1_ESM.docx]

**Supplementary Table 1.** Previous Literature on Disparities in Surgical Treatment for LSS

| **Study** | **Reference Number** | **Relevant Findings** |
| --- | --- | --- |
| Skolasky, R.L., et al. (2013) | 8 | The rate of surgical hospitalization among non-Hispanic White patients with LSS was significantly higher than that of either non-Hispanic Black or Hispanic patients with LSS. |
| Chen, Q., et al. (2023) | 9 | Black patients were significantly less likely than White patients to undergo spinal surgery to treat low back pain. |
| Aladdin, D.E.H., et al. (2020) | 10 | Post-operatively, Black patients had longer LOS and were more likely to experience complications and readmissions following inpatient lumbar spine surgery compared to White patients. |
| Lad, S.P., et al. (2013) | 11 | African-American patients were more likely than non-Hispanic White patients to experience postoperative complications and have longer LOS post-laminectomy or fusion for LSS. |
| Sanford, Z., et al. (2019) | 12 | African American patients experienced longer LOS and higher rates of complications than Caucasian patients after spine surgery. |
| Hagan, M.J., et al. (2022) | 13 | Patients in the most disadvantaged ADI quintile had a significantly increased chance of an extended LOS following lumbar surgery without fusion. |
| Holbert, S.E., et al. (2022) | 14 | Financial strain was associated with longer LOS among patients who underwent lumbar spine surgery. |
| Lad, S.P., et al. (2013) | 15 | Medicaid patients had significantly lower LSS surgery reoperation rates at 2 and more than 2 years compared to commercially insured patients. |

Abbreviations: LSS=lumbar spinal stenosis; LOS=length of stay; ADI=Area of Deprivation Index

| **Supplementary Table 2.** Codes Used to Identify Spine Interventions | |
| --- | --- |
|  |  |
| **Spine Intervention** | **Codes** |
| Coflex | CPT: 22867, 22868  ICD-10: 00NY0ZZ, 0SB00ZZ, 0SB20ZZ |
| Disc Procedure | CPT: 22857, 22858, 22862, 22865  ICD-10: 0RR50JZ, 0RW50JZ, 0SP20JZ, 0SP40JZ, 0SR20JZ, 0SR40JZ |
| Discectomy | CPT: 63076, 63077, 63078  ICD-10: 00NX0ZZ, 01N80ZZ |
| Drug Delivery Implant | CPT: 11981, 11982, 11983, 20702, 20703, 62350, 62351, 62362, 95990, 95991, 96522  ICD-10: 00WU33Z, 00WV33Z, 0JHT03Z, 0JHV03Z, 0JHW03Z, 0JPT03Z, 0JPV03Z, 0JPW03Z, 0JWT3VZ, 0JWV3VZ, 0JWW3VZ, 3C1ZX8Z, 00HU33Z, 0JH80VZ |
| Endoscopic Decompression | CPT: 62380  ICD-10: 00NY4ZZ, 01NB4ZZ |
| Fusion | CPT: 22558, 22585, 22612, 22614, 22630, 22632, 22633, 22634  ICD-10: 0SG0070, 0SG0071, 0SG007J, 0SG00A0, 0SG00AJ, 0SG00J0, 0SG00J1, 0SG00JJ, 0SG00K0, 0SG1070, 0SG107J, 0SG10A0, 0SG10AJ, 0SG10J0, 0SG10J1, 0SG10JJ, 0SG10K0, 0SG10K1, 0SG10KJ, 0SG30K0 |
| Interspinous Spacer | CPT: 0171T, 0172T |
| Kyphoplasty | CPT: 22510, 22511, 22512  ICD-10: 0PU43JZ, 0QU03JZ |
| Laminectomy with Fusion | CPT: 63052, 63053 |
| Laminectomy | CPT: 63005, 63017, 63030, 63035, 63042, 63043, 63044, 63047, 63048  ICD-10: 00NY0ZZ, 01NB0ZZ |
| MILD | CPT: 0275T  ICD-10: 00NY3ZZ |
| Neurostimulation | CPT: 63650, 63655, 63661, 63662, 63663, 63664, 63685, 63688, 95970, 95971, 95972  ICD-10: 00HV0MZ, 00HV3MZ, 00PV0MZ, 00WV0MZ, 00WV3MZ, 0PB40ZZ, 0QB00ZZ, 0QBS0ZZ, 4B00XVZ, 4B01XVZ |
| Removal of Implant | CPT: 22850, 22852, 22855  ICD-10: 0PP404Z, 0QP004Z, 0QP104Z |
| Repair Dural Cerebrospinal Fluid Leak | CPT: 62100, 63707, 63709  ICD-10: 00Q20ZZ, 00U10JZ, 00U10KZ, 00U207Z, 00U20KZ, 0JB00ZZ |
| Vertebral Excision | CPT: 63301, 63302, 63303, 63305, 63306, 63307, 63308  ICD-10: 00B20ZZ, 00BX0ZZ, 00BY0ZZ |
| Vertiflex | CPT: 22869, 22870  ICD-10: 0SH00BZ |
| Vertebroplasty | CPT: 22510, 22511, 22512  ICD-10: 0PU437Z, 0PU43JZ, 0PU43KZ, 0QU037Z, 0QU03JZ, 0QU03KZ, 0QU137Z, 0QU13JZ, 0QU13KZ |

Abbreviations: CPT=Current Procedural Terminology; ICD-10=International Classification of Diseases, 10th Revision; MILD=Minimally Invasive Lumbar Decompression
